# AMPK and Insulin Action - Responses to Ageing and High Fat Diet

**DOI:** 10.1371/journal.pone.0062338

**Published:** 2013-05-06

**Authors:** Christian Frøsig, Thomas E. Jensen, Jacob Jeppesen, Christian Pehmøller, Jonas T. Treebak, Stine J. Maarbjerg, Jonas M. Kristensen, Lykke Sylow, Thomas J. Alsted, Peter Schjerling, Bente Kiens, Jørgen F. P. Wojtaszewski, Erik A. Richter

**Affiliations:** 1 Section of Molecular Physiology, The August Krogh Centre, Department of Nutrition, Exercise and Sports, University of Copenhagen, Copenhagen, Denmark; 2 Institute of Sports Medicine, Department of Orthopedic Surgery M, Bispebjerg Hospital and Center for Healthy Aging, Faculty of Health Sciences, University of Copenhagen, Copenhagen, Denmark; Oregon Health & Science University, United States of America

## Abstract

The 5′-AMP-activated protein kinase (AMPK) is considered “a metabolic master-switch” in skeletal muscle reducing ATP- consuming processes whilst stimulating ATP regeneration. Within recent years, AMPK has also been proposed as a potential target to attenuate insulin resistance, although the exact role of AMPK is not well understood. Here we hypothesized that mice lacking α2AMPK activity in muscle would be more susceptible to develop insulin resistance associated with ageing alone or in combination with high fat diet. Young (∼4 month) or old (∼18 month) wild type and muscle specific α2AMPK kinase-dead mice on chow diet as well as old mice on 17 weeks of high fat diet were studied for whole body glucose homeostasis (OGTT, ITT and HOMA-IR), insulin signaling and insulin-stimulated glucose uptake in muscle. We demonstrate that high fat diet in old mice results in impaired glucose homeostasis and insulin stimulated glucose uptake in both the soleus and extensor digitorum longus muscle, coinciding with reduced insulin signaling at the level of Akt (pSer473 and pThr308), TBC1D1 (pThr590) and TBC1D4 (pThr642). In contrast to our hypothesis, the impact of ageing and high fat diet on insulin action was not worsened in mice lacking functional α2AMPK in muscle. It is concluded that α2AMPK deficiency in mouse skeletal muscle does not cause muscle insulin resistance in young and old mice and does not exacerbate obesity-induced insulin resistance in old mice suggesting that decreased α2AMPK activity does not increase susceptibility for insulin resistance in skeletal muscle.

## Introduction

Insulin resistance in peripheral tissues is a hallmark characteristic of obesity-related type 2 diabetes (T2D). In this context, skeletal muscle is a critical organ constituting ∼40% of body weight and contributing the majority of whole body insulin-stimulated glucose disposal [1]. Insulin resistance, associated with high fat feeding or obesity, is believed to be the combined result of chronic low-grade inflammation and accumulation of bio-active lipid species within muscle. In turn, this leads to impairment of insulin signaling to GLUT4 translocation and subsequently insulin-stimulated glucose uptake [2], [3]. Consistent with a pathogenic role of intracellular lipid accumulation, genetically engineered mouse models with improved capacity for lipid oxidation in muscle are protected against adverse effects of high fat feeding [4], [5]. The AMP-activated protein kinase (AMPK) is activated in response to stimuli that increase the intracellular ratio of AMP/ATP, such as exercise, hypoxia, osmotic stress and ischemia [6]–[8]. AMPK is referred to as “a metabolic master-switch” due to its general ability to reduce ATP consuming anabolic processes while alternative pathways for ATP regeneration are stimulated [9], including stimulation of lipid oxidation in muscle [10], [11]. This has led to the speculation that AMPK activation may protect muscle from high fat feeding induced insulin resistance. To support this, Goodyear et al. demonstrated that overexpression of kinase dead α2AMPK in mice on a FVB mouse background (AMPK Ki), resulted in massive exacerbation of muscle insulin resistance in response to 30 weeks of high fat feeding, coinciding with an increase in muscle diacylglycerol content [12]. Interestingly, the AMPK Ki mice on chow diet in that study also showed a tendency towards impaired insulin action (∼50% reduction vs. wild type (WT), p = 0.07) whereas normal insulin action has previously been reported in AMPK Ki mice after only 15 weeks of chow diet [13]. This suggests that acute lack of α2AMPK activation may not directly trigger impaired insulin action, but rather lack of α2AMPK activity over time leads to a muscle phenotype that is more susceptible for insulin resistance. This would also explain our previous observation that insulin resistance induced by 12 weeks high fat feeding was not exacerbated in young kinase dead α2AMPK mice on a C57BL/6J background (AMPK KD) [14].

In order to test this interpretation and firmly link AMPK with muscle insulin action, we here hypothesized that old AMPK KD mice would develop insulin resistance on a chow diet (as indicated by observations in AMPK Ki mice). Furthermore, we hypothesized that insulin resistance induced by high fat feeding would be exacerbated in old AMPK KD mice (in contrast to observations young AMPK KD mice).

## Materials and Methods

### Ethics

All animal experiments were approved by the Danish Animal Experimental Inspectorate (# 2012-15-2934-00310) in compliance with the European Convention for Protection of Vertebrate Animals Used for Scientific Purposes. All efforts were made to minimize suffering during in vivo experiments. Furthermore, surgery was performed under sodium pentobarbital anesthesia and after surgery animals were sacrificed by cervical dislocation.

### Animals

Animals used were age 4.2±0.1 month (Young) or 18.0±0.2 month (Old) C57BL/6J male mice overexpressing a muscle-specific, kinase-dead α2AMPK construct (AMPK KD), as described by Mu et al. [15], [16] and corresponding WT littermates. Briefly, the animals overexpress a Lys45-to-Arg mutant of the α2AMPK protein, driven by a heart- and skeletal muscle-specific creatine kinase promoter. Average age.

### Diet Treatments

Animals were kept on a 10∶14-h light-dark cycle with unlimited access to standard rodent diet (60% [of energy] carbohydrates, 27% protein and 13% fat) and water (CHO groups). In the high fat diet group (FA group) standard chow was replaced 17 weeks prior to terminal experiments with a high fat diet (20% [of energy] carbohydrates, 20% protein and 60% fat) specified as 19.7% [of energy] casein, 54.4% lard, 5.5% soybean oil, 12.3% maltodextrin and 6.7% sucrose (#D12492; Research diets, Inc, New Brunswick, NJ, USA).

### Whole Body Glucose Homeostasis

Glucose (OGTT) and insulin (ITT) tolerance tests were performed after a 5 hours fast on separate occasions in all mice, 2–3 weeks prior to terminal experiments. Glucose (2 g/kg body weight) was given by oral gavage and insulin (0.5 units/kg body wt; Actrapid, Novo Nordisk, Bagsværd, Denmark) was given intraperitoneally at time 0 min. Blood samples were collected from the tail at different time points (−15, 20, 40, 60, 90 and 120 min). Blood glucose was measured using a glucometer (Bayer, Leverkusen, Germany), and area under the curve (AUC) was calculated as a weighted average of all glucose measurements. In blood samples obtained at −15 min and 20 min in the OGTT, plasma insulin was determined using a two-site enzyme immunoassay (DRG instruments, Marburg, Germany). Based on basal glucose and insulin measurements, homeostatic model assessment of insulin resistance (HOMA-IR) was calculated as HOMA-IR = [glucose (mg/dl)×insulin (µU/ml)]/405 [17].

### Body Composition and Metabolic Measurements

1–2 weeks prior to terminal experiments, all mice were weighed, and scanned for body composition (EchoMRI-4in1; EchoMRI, Houston, TX, USA). Following 48 hours of acclimatization to individual metabolic cages with access to food (either CHO or FA diet) and water ad libitum, the metabolic cages were sealed and O_2_ uptake and CO_2_ production were measured for 24 hours (Fed conditions) using a CaloSys apparatus (TSE Systems GmbH, Bad Homburg, Germany). Next, food was removed from the cages (Fasted conditions) and similar measurements were made for an additional 24 hours. Data for VO_2_ and RER (VCO_2_/VO_2_) was calculated as AUC using a weighted average of time points obtained every hour. Following these measurements mice were returned to standard cages and were housed individually for at least one week prior to terminal experiments.

### Muscle Incubation and Glucose Uptake

On the day of terminal experiments, animals were anesthetized intraperitoneally by injection of pentobarbital sodium (6 mg/100 g body wt). In all mice, m. Soleus (SOL) and m. Extensor Digitorum Longus (EDL) were quickly excised and suspended in incubation chambers (Multi Myograph system; Danish Myo-Technology, Aarhus, DK). Muscles were incubated for 40 min in prebuffer (standard Krebs-Henseleit-Ringer buffer with addition of 8 mM Mannitol, 2 mM pyruvate, and 0.01% BSA) at 30°C and oxygenated with a gas mixture containing 95% O_2_ and 5% CO_2_. Subsequently, muscles were incubated in stimulation buffer (standard Krebs-Henseleit-Ringer buffer with addition of 7 mM Mannitol, 1 mM 2-deoxy-D-glucose (2DG) and 0.01% BSA) containing 500 µU/ml (3 nM) insulin (Actrapid; Novo Nordisk, Bagsvaerd, Denmark) for 10 min and next for an additional 10 min in stimulation buffer with insulin and tracers (0.75 µCi/ml 2-[2,6-^3^H]deoxy-D-glucose and 0.32 µCi/ml [1-^14^C]mannitol [PerkinElmer, MS, USA]). After incubation, muscles were harvested, washed in ice-cold Krebs-Henseleit buffer, blotted on filter paper and frozen in liquid N_2_ for later analyses.

### Muscle Lysate Preparation

SOL and EDL muscles were dissected free of tendons and homogenized in ice-cold buffer (10% glycerol, 20 mM sodium pyrophosphate, 1% NP-40, 2 mM PMSF, 150 mM sodium chloride, 50 mM Hepes, 20 mM β-glycerophosphate, 10 mM sodium fluoride, 1 mM EDTA, 1 mM EGTA, 10 µg/ml aprotinin, 3 mM benzamidine, 10 µg/ml leupeptin and 2 mM sodium orthovanadate (pH 7.4)). Homogenates were subsequently rotated end-over-end for 1 hour at 4°C before being centrifuged at 16,000 g at 4°C for 25 min. Supernatants were collected and stored at −80°C for later analyses. Total protein concentrations were determined in triplicates with a coefficient of variance maximum of 5% by the bicinchoninic acid method (Pierce Biotechnology, Rockford, IL, USA).

### 2-deoxy-D-glucose (2DG) Uptake

2DG uptake was measured by mixing 150 ul muscle lysate protein (∼600 ug) in 3 ml scintillation fluid (Ultima Gold; PerkinElmer, Waltham, MS, USA). Subsequently radioactivity was measured by liquid scintillation counting (Tri-Carb 2910; PerkinElmer, Waltham, MS, USA). Prior to this procedure, it was verified that the homogenization buffer did not interfere with measurements of radioactivity (data not shown).

### SDS-PAGE and Western Blot Analyses

For analyses of Akt Ser473, Akt Thr308, TBC1D4 Thr642, and TBC1D1 Thr590 phosphorylation as well as protein content of GLUT4, HK2, and TRB-3, aliquots of muscle lysate (20–30 µg) was heated in SDS sample buffer (5 min, 96°C) and loaded onto gels. Muscle proteins were separated using 5%, 7.5% or 10% Tris-HCl gels (Bio-Rad Laboratories, Copenhagen, DK), and transferred (semidry) to PVDF membranes (Immobilon Transfer Membrane, Millipore, Glostrup, DK). After blocking (TBS +0.05% Tween20 (TBST) +2% skim milk powder) the membranes were incubated with primary antibodies (TBST +2% skim milk powder) followed by incubation with horseradish peroxidase-conjugated secondary antibodies. After detection (Kodak Digital Science Image Station 2000 M, Model: 440CF, Eastman Kodak Company, USA) and quantification by densitometry software (Eastman Kodak Company USA) the signal was finally corrected for between gel variation relative to a muscle lysate standard run in duplicate on all gels. Membranes used for detection of Akt Ser473, Akt Thr308, TBC1D4 Thr642 and TBC1D1 Thr590 phosphorylation were subsequently stripped (100 mM 2-mercaptoethanol, 2% SDS, and 62.5 mM Tris-HCl) for 2 h at 50°C before being reprobed with antibodies targeting Akt2, TBC1D4 and TBC1D1 protein, respectively. This allowed for expression of phosphorylation relative to total protein content on the same membrane.

### Antibodies

The following antibodies were used in this study. Akt Ser473, TBC1D1 Thr590, Akt2 and HK2 antibodies were from Cell signaling technologies (Danvers, MA, USA). Akt Thr308 and TBC1D4 were from Upstate biotechnology (Lake Placid, NY, USA). The TBC1D4 Thr642 antibody was from Symansis (Auckland, New Zealand), GLUT4 from Thermo scientific (Rockford, IL, USA) and the TBC1D1 antibody was generated as previously described [18].

### Statistics

Data are expressed as means ± SE. Values in young vs. old mice on CHO diet were compared by two-way (for results without insulin stimulation) or three-way (for results with insulin stimulation) analysis of variance (ANOVA). Similarly, values in old mice on CHO diet vs. old mice on FA diet were compared by two or three-way analysis of variance (ANOVA). If analysis of variance revealed significant interactions, a Tukey’s post hoc test for multiple comparisons was performed. P values below 0.05 were considered statistically significant.

## Results

### Body Composition and Metabolic Characterization

As reported in Table 1, body composition (body weight and fat free mass) and metabolic adaptation (VO_2_ and RER) to feeding and fasting were similar in young WT and AMPK KD mice. When compared to young mice, old mice on CHO diet had increased body weight (∼15%, p<0.05) and fat free mass (∼10%, p<0.05), whereas metabolic characteristics were similar (NS). In old mice maintained on a FA diet for 17 weeks, a further increase (∼30%, p<0.01) in body weight was observed whereas fat free mass slightly decreased (∼5%, p<0.01). This is consistent with a direct effect of the FA diet on body fat accumulation. The FA diet also led to an expected decreased RER in both the fed (∼−20%, p<0.01) and fasted (∼−15%, p<0.01) state whereas VO_2_ was unaltered (NS). Interestingly, old AMPK KD mice were slightly smaller (∼5%, p<0.05) than old WT mice independent of diet, as indicated by both reduced body weight and fat free mass.

**Table 1:** Body composition and metabolic characterization.

	Young CHO		Old CHO		Old FA	
	WT	AMPK KD	WT	AMPK KD	WT	AMPK KD
Body weight (g)	33.5±0.8	32.9±0.5	39.1±1.0‡	38.1±0.6‡ ^$^	53.0±1.7†	48.6±1.6† ^$^
Fat free mass (g)	29.6±0.9	30.2±0.5	32.8±0.8‡	32.0±0.7‡ ^$^	31.8±0.7†	29.6±0.6† ^$^
VO2 (ml/hr/kg), Fed	2095±160	2278±143	2061±97	2289±164	2263±89	2334±113
VO2 (ml/hr/kg), Fasted	1945±44#	1958±57#	1848±57#	1995±75#	1958±79#	2115±112#
RER, Fed	0.97±0.05	0,94±0.03	0,97±0.04	0.96±0.04	0.80±0,01†	0.81±0.01†
RER, Fasted	0.85±0.04#	0,84±0.03#	0.84±0.02#	0.84±0.02#	0.72±0.00^#^ †	0.73±0.01^#^ †

Body composition, VO2 and RER were determined in young and old AMPK KD mice and WT littermates on chow diet (CHO) or in old mice after 17 weeks of high fat diet (FA).

‡ Main effect of fasting vs. fed conditions, p<0.001.

# Main effect of diet, p<0.01.

$ Main effect of age, p<0.005.

† Main effect of genotype, p<0.05. Values are means ± SE. n = 7–16.

### Whole Body Glucose Homeostasis

In order to investigate the effect of genotype, age and diet on whole body glucose homeostasis, mice underwent an OGTT and an ITT. Before and after 20 min in the OGTT plasma insulin concentrations were measured and HOMA-IR index was calculated based on basal values.

### OGTT Glucose and Insulin Values

**Figure 1:**
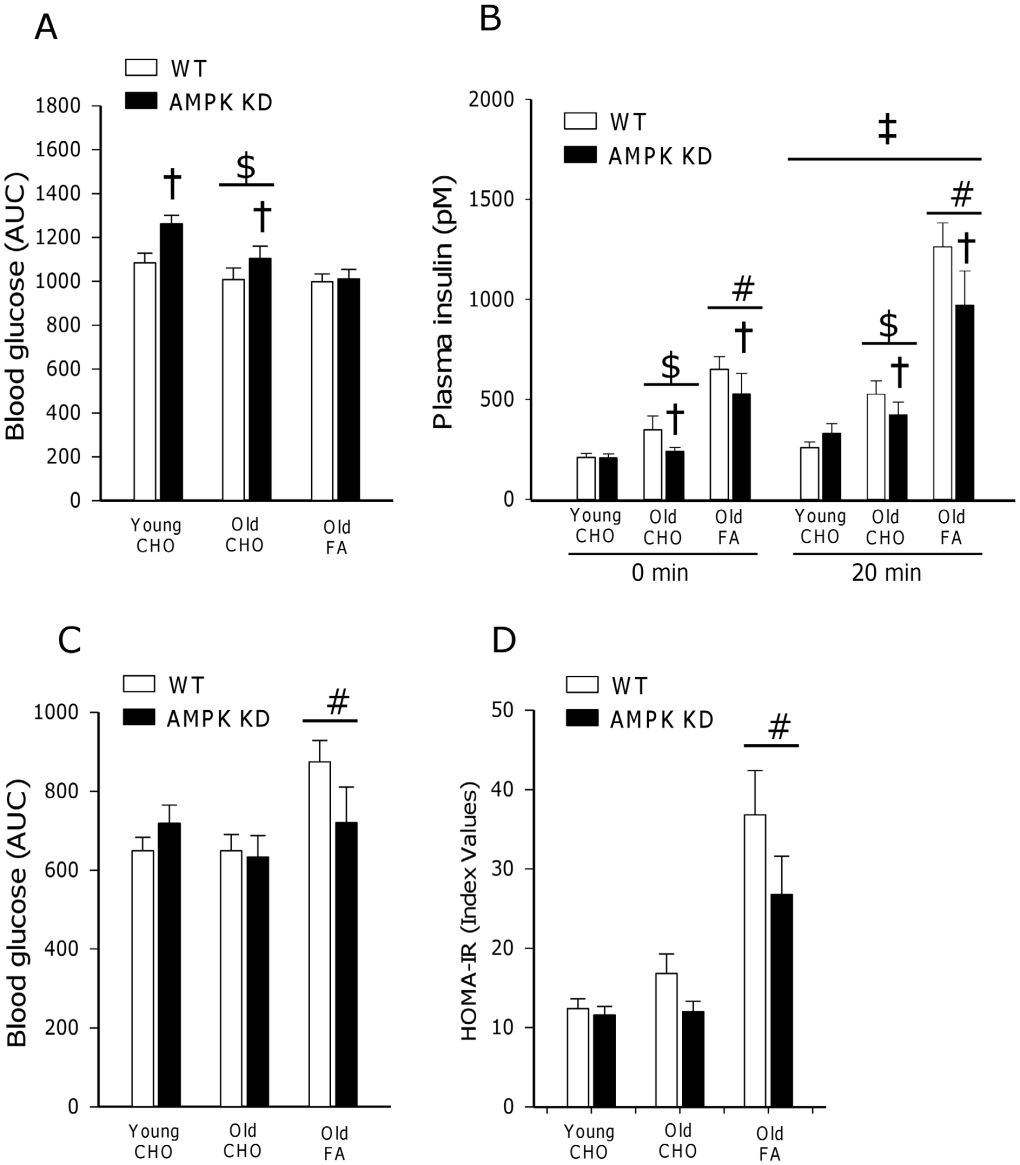
Characterization of whole body glucose homeostasis. (A) OGGT (2 g/kg body weight) and C) ITT (0.5 U/kg body weight) were conducted after 5 hours of fasting. Values are expressed as AUC based on weighted means of all glucose measurements (t = −15, 20, 40, 60, 90 and 120 min). Figure 1B shows plasma insulin concentrations before (0 min) and after 20 min in response to the OGTT. Figure 1D illustrates calculated HOMA-IR index values based on basal glucose and insulin concentrations obtained after 5 hour of fasting. Measurements were made in young and old AMPK KD mice and WT littermates on chow diet (CHO) or in old mice after 17 weeks of high fat diet (FA). $: Main effect of age, p<0.001. #: Main effect of diet, p<0,001. †: Main effect of genotype, p<0.005. ‡: Main effect of time, p<0.001. Values are means ± SE. n = 11–17.

When comparing mice on CHO diet, glucose AUC in response to the OGTT was reduced (∼−10%, p<0.001) with age but was increased (∼10%, p<0.005) in both young and old AMPK KD mice compared to WT (Figure 1A). This should be seen in context of increased (∼30%, p<0.05) insulin concentrations in old mice as well as decreased (∼−20%, p<0.05) insulin concentrations in old AMPK KD mice on CHO diet (Figure 1B). In response to the FA diet, glucose AUC in response to the OGTT was unaltered (NS), however the FA diet resulted in a marked increase (∼100%, p<0.001) in insulin concentrations prior to and during the OGTT (Figure 1A and 1B). Furthermore, in old AMPK KD mice, both basal and 20 min insulin concentrations were reduced (∼−15%, p<0.05) after the FA diet similar to the observations after the CHO diet (Figure 1B).

### ITT Glucose Values

*G*lucose AUC in response to the ITT was not influenced by genotype and did not change with ageing in mice on the CHO diet. However, old mice on FA diet were characterized by increased (∼20%, p<0.001) glucose AUC in response to the ITT when compared to the CHO diet (Figure 1C).

### HOMA-IR Index Values

HOMA-IR index values were not influenced by genotype and did not change with ageing after the CHO diet. In contrast, old mice on FA diet were characterized by increased (∼100%, p<0.001) HOMA-IR index values when compared to the CHO diet (Figure 1D).

### Muscle Insulin-stimulated Glucose Uptake

**Figure 2:**
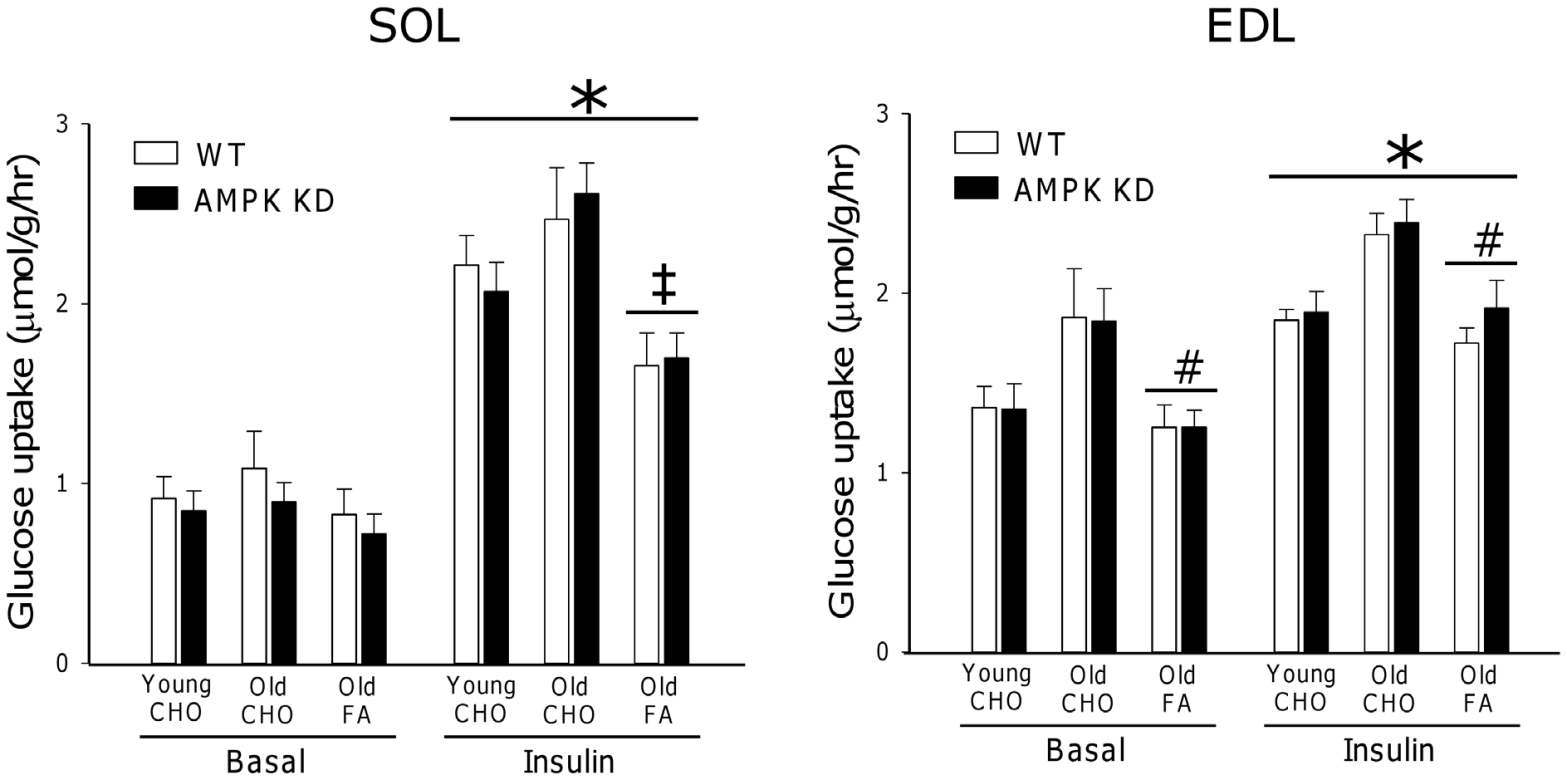
*In vitro* glucose uptake. Basal (0 µU/ml) and insulin (500 µU/ml) stimulated glucose uptake measured *in vitro* in m. Soleus (SOL) and m. Extensor Digitorum Longus (EDL). Measurements were made in young and old AMPK KD mice and WT littermates on chow diet (CHO) or in old mice after 17 weeks of high fat diet (FA). *: Main effect of insulin, p<0.001. #: Main effect of diet, p<0,001. ‡: Interaction between diet and insulin action, p<0.001. Values are means ± SE. n = 11–15.

To investigate the role of genotype, age and diet on muscle glucose uptake, insulin-stimulated glucose uptake was measured in SOL and EDL muscles *in vitro* in response to 500 µU/ml insulin. Compared to a subset of muscles stimulated with 10.000 µU/ml; 500 µU/ml was verified as a sub-maximal insulin stimulus leading to ∼70% of maximal insulin-stimulated glucose uptake in both muscles (data not shown). In SOL, insulin stimulation resulted in a ∼150% increase (p<0.001) in glucose uptake in both young and old mice on CHO diet when compared to basal. In contrast, in old mice on the FA diet, the insulin response was significantly impaired, corresponding to ∼65% (p<0.001) of values in old mice on CHO diet (Figure 2). In EDL, insulin-stimulated glucose uptake was increased by ∼40%, (p<0.001) in young and old mice on CHO diet when compared to basal. Although a trend towards generally greater glucose uptake in the old mice was observed, this did not reach statistical significance (p = 0.09). In contrast, glucose uptake in old mice on FA diet was decreased by ∼30% (p<0.001) compared with old mice on CHO diet, but the response to insulin was preserved (Figure 2). Notably, in both SOL and EDL muscles, basal and insulin-stimulated glucose uptake in all groups was independent of genotype (NS).

### Muscle Insulin Signaling

**Figure 3:**
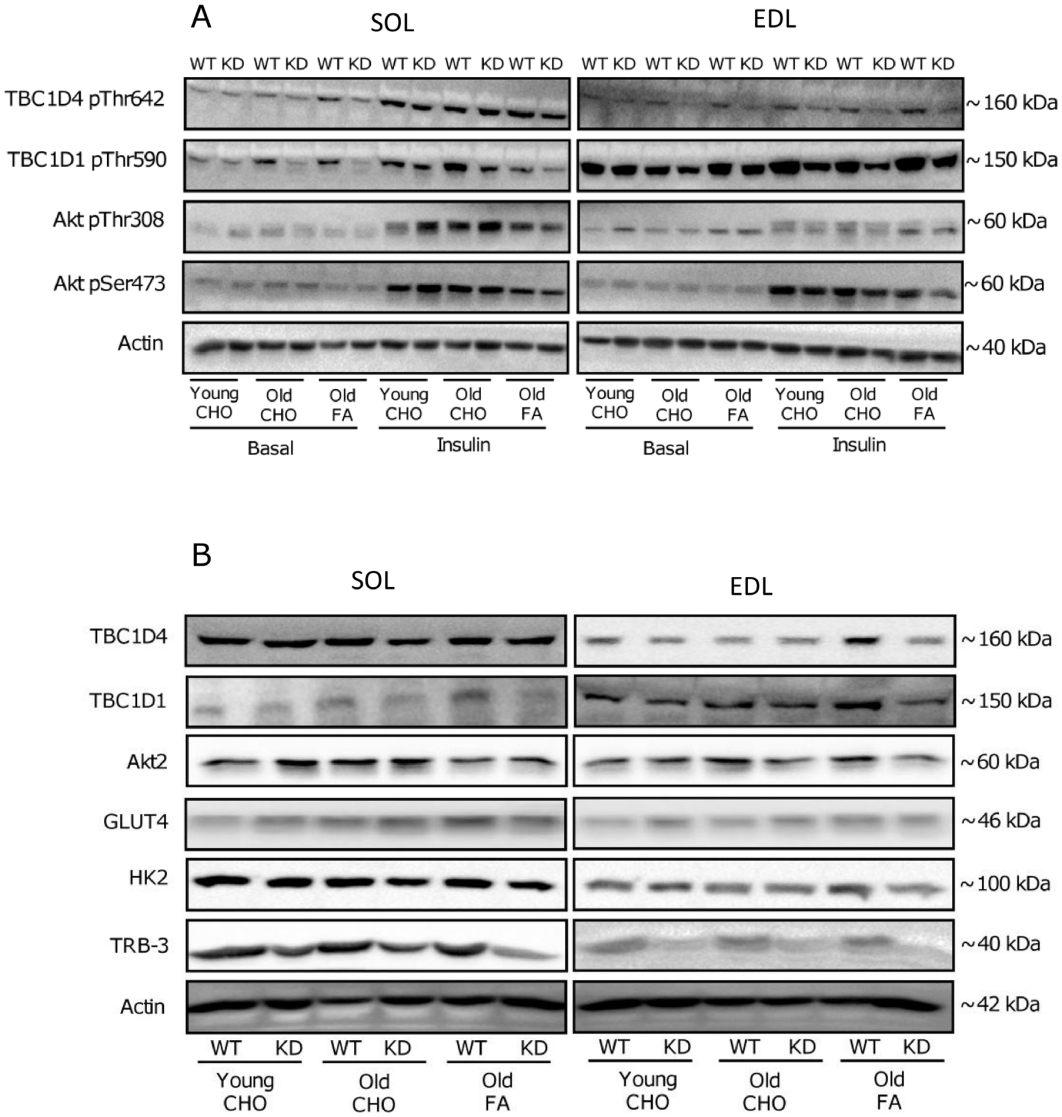
Representative immuno-blots. Representative immuno-blots of protein phosphorylations (A) and total protein expression (B) measured by Western blot analyses in muscle lysates. Left columns are images from m. Soleus (SOL) and right columns are images from m. Extensor Digitorum Longus (EDL). Specification of phosphorylation sites and proteins evaluated are indicated on the left and estimated molecular migration points (KDa) on the gels are indicated on the right. In Figure 3A, basal and insulin-stimulated protein phosphorylations are illustrated in young and old AMPK KD mice and WT littermates on chow diet (CHO) and in old mice after 17 weeks of high fat diet (FA). Figure 3B illustrates representative images of protein expression in basal samples.

To elucidate, if adaptations in insulin-stimulated glucose uptake reflected on muscle insulin signaling, phosphorylation of Akt, TBC1D1 and TBC1D4 was evaluated by western blotting. Representative blots can be viewed in Figure 3A and 3B.

### Akt Phosphorylation

**Figure 4:**
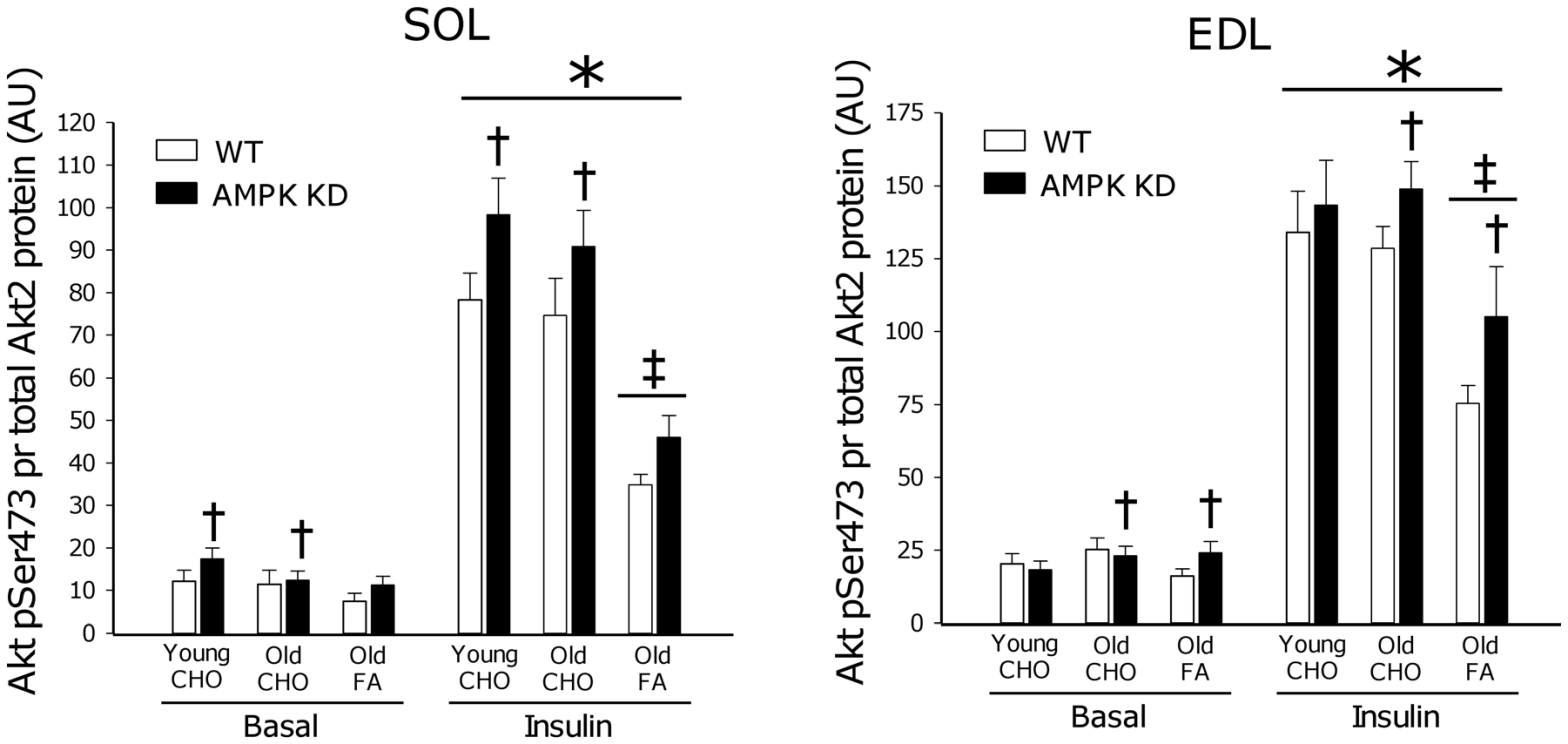
Akt Ser473 phosphorylation. Basal (0 µU/ml) and insulin (500 µU/ml) stimulated Akt Ser473 phosphorylation measured by Western blot analyses in m. Soleus (SOL) and m. Extensor Digitorum Longus (EDL). Measurements were made in young and old AMPK KD mice and WT littermates on chow diet (CHO) or in old mice after 17 weeks of high fat diet (FA). *: Main effect of insulin, p<0.001. ‡: Interaction between diet and insulin action, p<0.001. †: Main effect of genotype in either CHO fed or Old groups, p<0.05. Values are means ± SE. n = 11–15.

**Figure 5:**
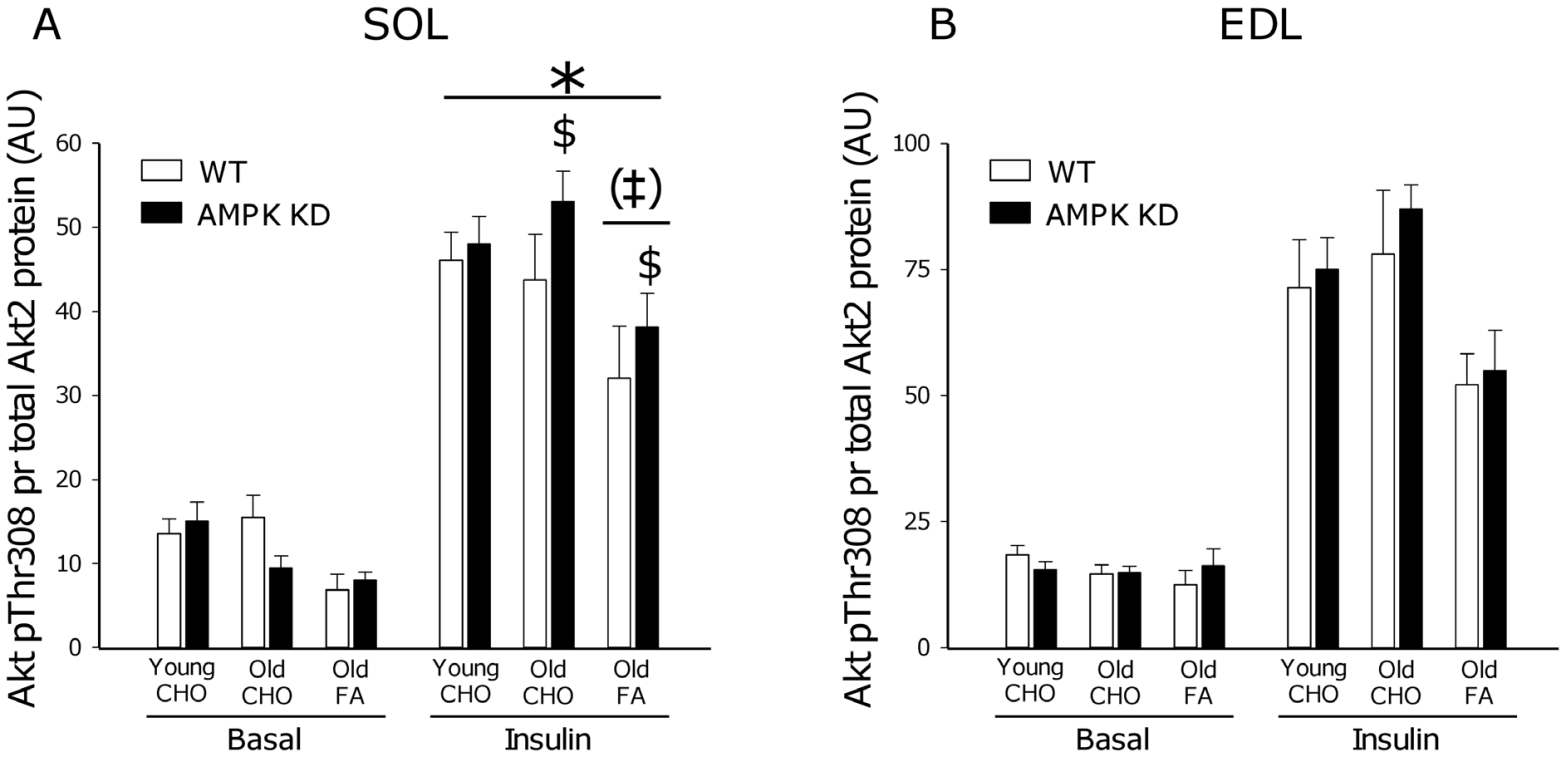
Akt Thr308 phosphorylation. Basal (0 µU/ml) and insulin (500 µU/ml) stimulated Akt Thr308 phosphorylation measured by Western blot analyses in m. Soleus (SOL) and m. Extensor Digitorum Longus (EDL). Measurements were made in young and old AMPK KD mice and WT littermates on chow diet (CHO) or in old mice after 17 weeks of high fat diet (FA). *: Main effect of insulin, p<0.001. ‡: Interaction between diet and insulin action, p<0.001. (‡): Trend towards interaction between diet and insulin action, p = 0.06. $: Interaction between genotype and insulin action, p<0.05. Values are means ± SE. n = 11–15.

**Figure 8:**
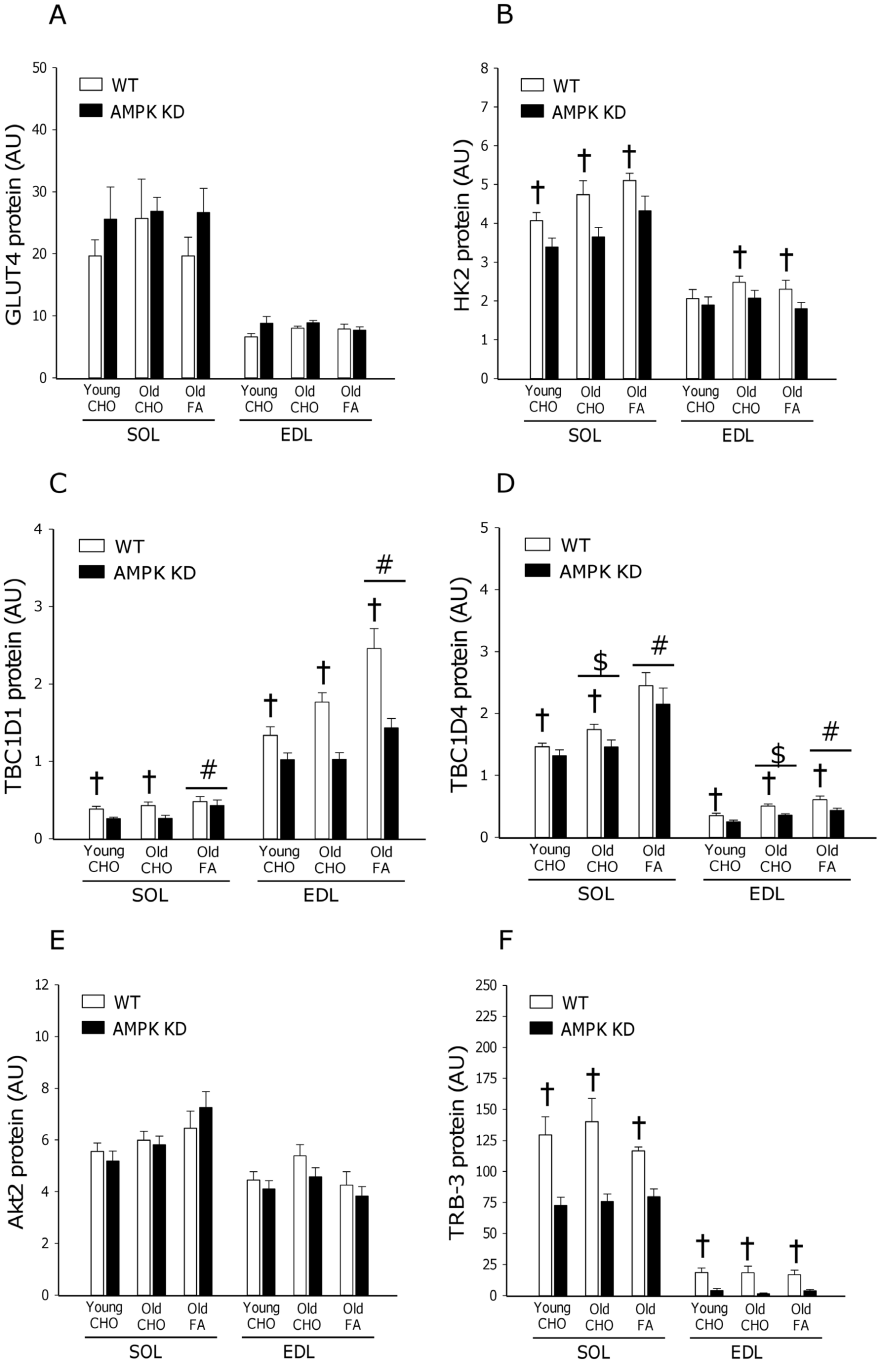
Protein content of GLUT4, HK2, TBC1D1, TBC1D4, Akt2 and TRB-3. Protein content of GLUT4 (A), HK2 (B), TBC1D1(C), TBC1D4 (D), Akt2 (E) and TRB-3 (F) was measured by Western blot analyses in basal muscle samples from m. Soleus (SOL) and m. Extensor Digitorum Longus (EDL). Measurements were made in young and old AMPK KD mice and WT littermates on chow diet (CHO) or in old mice after 17 weeks of high fat diet (FA). #: Main effect of diet, p<0.05. $: Main effect of age, p<0.05. †: Main effect of genotype, p<0.05. Values are means ± SE. n = 11–15.

Akt Ser473 and Thr308 phosphorylation are markers of Akt activity in skeletal muscle. When expressed per total Akt2 protein (the principal Akt isoform regulated by insulin in muscle [19]) a ∼300–400% increase (p<0.001) in phosphorylation was observed in both young and old mice on a CHO diet in response to insulin stimulation (Figure 4 and 5). Interestingly, when old mice were on FA diet, insulin-stimulated Akt phosphorylation (both Ser473 and Thr308) was reduced by ∼30% (p<0.001) in all muscles, except for Thr308 phosphorylation in SOL muscle where this was only observed as a trend (p = 0.06). In some, but not all muscles, Akt phosphorylation was modestly increased (∼10–20%) in AMPK KD mice when compared to WT. As illustrated in Figure 8C, adaptations in Akt phosphorylation cannot be ascribed to differences in protein content of Akt2.

### TBC1D4 Phosphorylation

**Figure 6:**
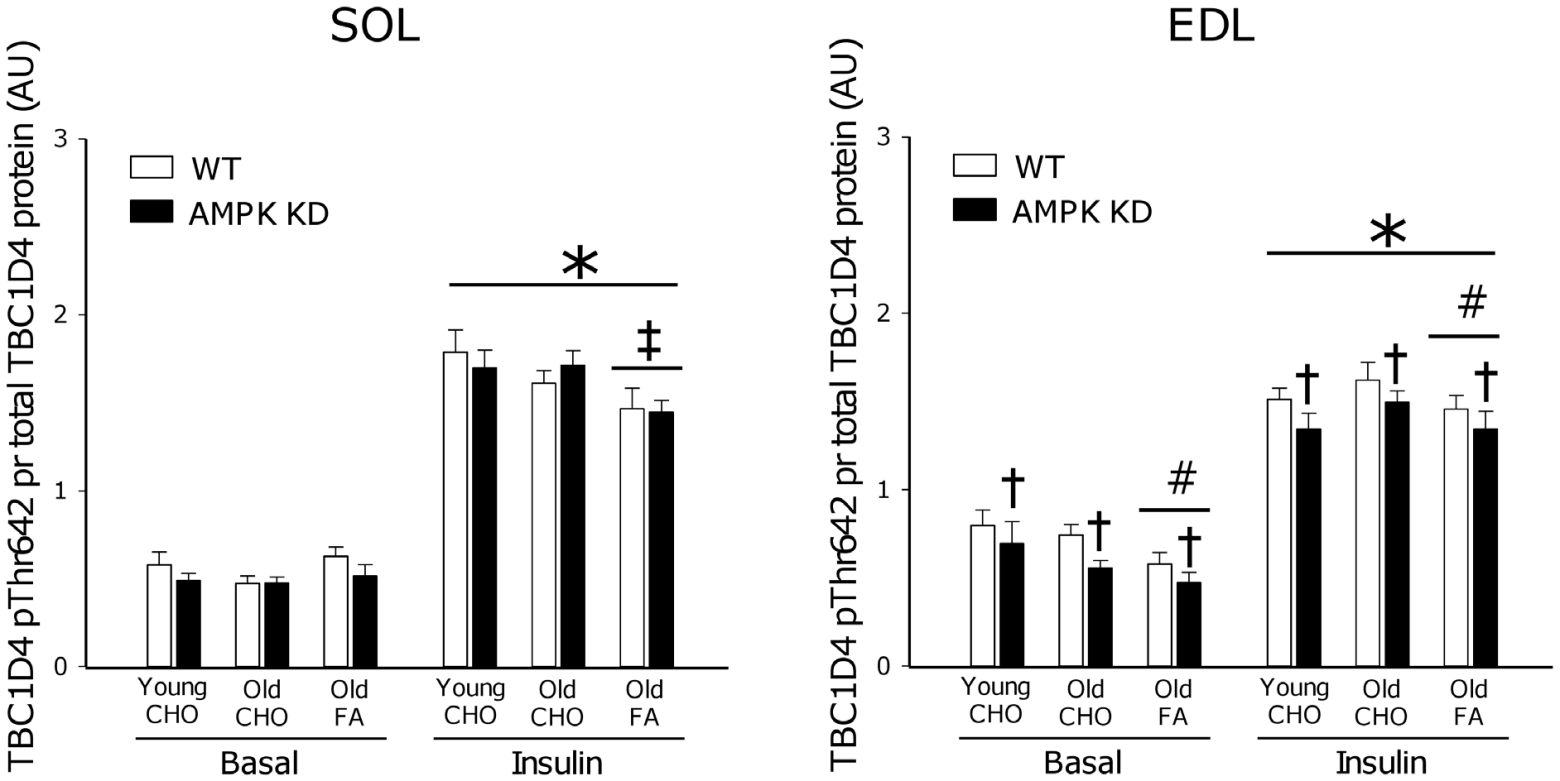
TBC1D4 Thr642 phosphorylation. Basal (0 µU/ml) and insulin (500 µU/ml) stimulated TBC1D4 Thr642 phosphorylation measured by Western blot analyses in m. Soleus (SOL) and m. Extensor Digitorum Longus (EDL). Measurements were made in young and old AMPK KD mice and WT littermates on chow diet (CHO) or in old mice after 17 weeks of high fat diet (FA). *: Main effect of insulin, p<0.001. †: Main effect of genotype, p<0.05. #: Main effect of diet, p<0.005. ‡: Interaction between diet and insulin action, p<0.001. Values are means ± SE. n = 11–15.

Thr642 phosphorylation on TBC1D4 is believed to inhibit TBC1D4 GAP function, as part of a signal facilitating GLUT4 translocation [20]. When expressed per total TBC1D4 protein, we observed a ∼150–200% increase (p<0.001) in phosphorylation of this site in response to insulin stimulation in both young and old mice on the CHO diet (Figure 6). In old mice, 17 weeks of FA diet reduced (∼−15%, p<0.001) insulin-stimulated TBC1D4 phosphorylation in SOL. Furthermore, in EDL muscle the FA diet reduced (∼−10%, p<0.005) both basal and insulin-stimulated TBC1D4 phosphorylation. AMPK KD mice were characterized by a modest (∼−10%, p<0.05) reduction in TBC1D4 phosphorylation in both basal and insulin-stimulated EDL muscle. In this context, it should be emphasized that TBC1D4 total protein was reduced (∼10–20%, p<0.05) in both SOL and EDL muscle of AMPK KD mice (Figure 8D) and interestingly TBC1D4 protein increased (∼10–20%, p<0.05) with age in both WT and AMPK KD mice. Finally, 17 weeks of FA diet in old mice resulted in an additional increment (∼20–30%, p<0.05) in TBC1D4 protein.

### TBC1D1 Phosphorylation

**Figure 7:**
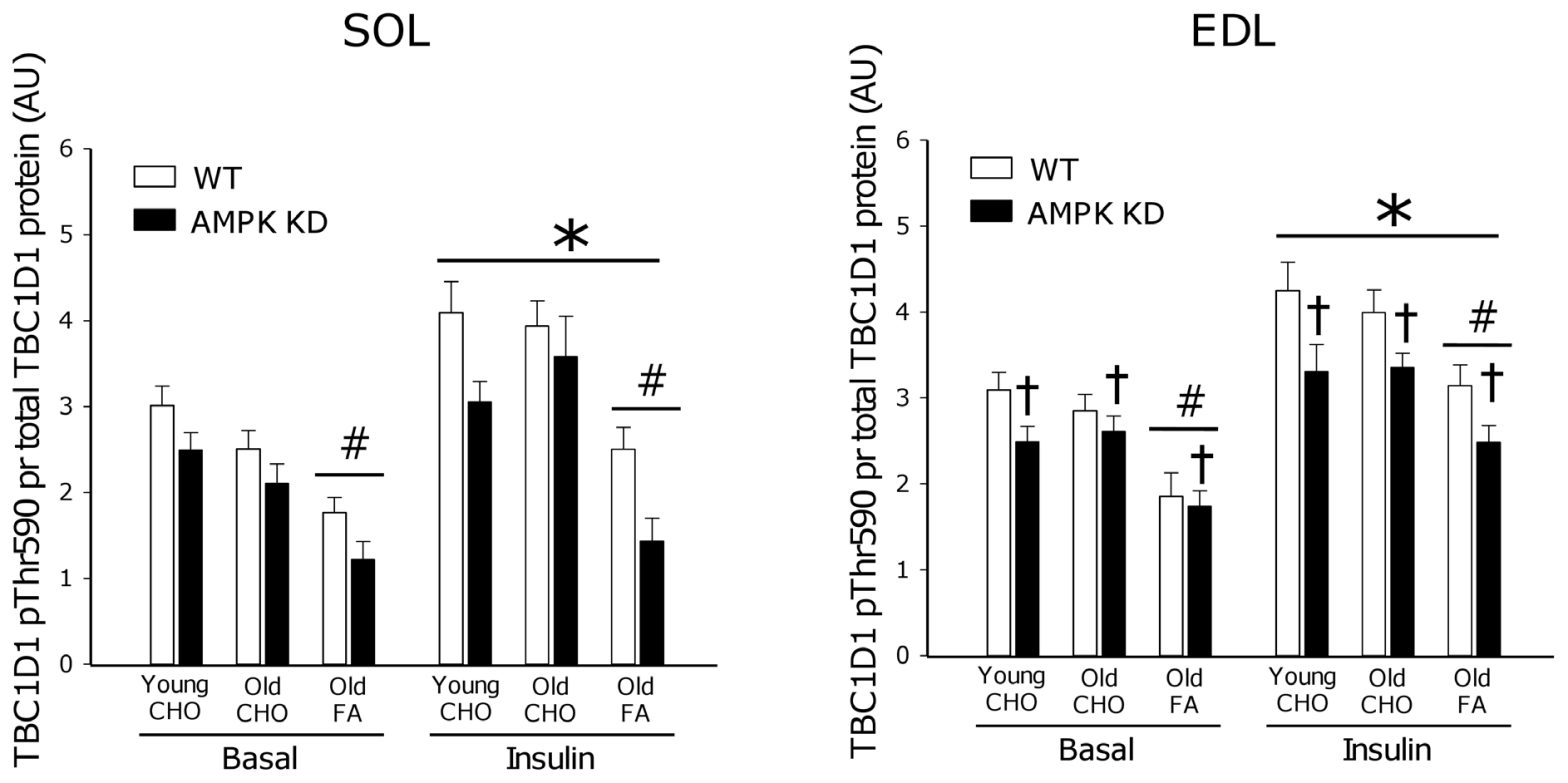
TBC1D1 Thr590 phosphorylation. Basal (0 µU/ml) and insulin (500 µU/ml) stimulated TBC1D1 Thr590 phosphorylation measured by Western blot analyses in m. Soleus (SOL) and m. Extensor Digitorum Longus (EDL). Measurements were made in young and old AMPK KD mice and WT littermates on chow diet (CHO) or in old mice after 17 weeks of high fat diet (FA). *: Main effect of insulin, p<0.001. #: Main effect of diet p<0.001. †: Main effect of genotype, p<0.05. Values are means ± SE. n = 11–15.

Complimentary to TBC1D4 although less well characterized, Thr590 phosphorylation on TBC1D1 is believed to inhibit TBC1D1 GAP function to allow for vesicle translocation [21]. When expressed per total TBC1D1 protein, we detected a ∼50% increase (p<0.001) in phosphorylation of Thr590 in response to insulin stimulation in both young and old mice on the CHO diet (Figure 7). Furthermore, in both SOL and EDL muscle, old mice on FA diet had reduced (∼−40%, p<0.001) TBC1D1 phosphorylation in basal and insulin-stimulated muscle and a modest reduction (∼15%, p<0.05) in TBC1D1 phosphorylation was observed in AMPK KD mice when compared to WT in EDL muscle. At the protein level, muscles of AMPK KD mice were characterized by markedly reduced (∼−40%, p<0.001) TBC1D1 protein content whereas TBC1D1 protein content increased (∼30%, p<0.05) in muscle of old mice after the FA diet (Figure 8C).

### Muscle Protein Expression

To further evaluate muscle proteins in relation to insulin action, we next measured protein content of GLUT4, HK2 and TRB-3 in basal SOL and EDL muscle (Figure 8A, 8B and 8F). These proteins were more heavily expressed in SOL compared to EDL, but neither changed in response to ageing nor with FA diet. However, protein content of both HK2 (∼−20%, p<0.05) and TRB-3 (∼−50–80%, p<0.001) was suppressed in AMPK KD mouse muscles.

## Discussion

In response to high fat feeding, mouse skeletal muscle rapidly (within weeks) becomes insulin resistant at the level of glucose uptake. In many, but not all, studies this is associated with impairment of insulin signaling to GLUT4 translocation, detectable at the level of IRS-1 associated PI-3 kinase [22]–[24], Akt [14], [24], [25] and aPKC [26]. In the present study we provide novel evidence linking high fat feeding to defects in insulin signaling to Akt (Figure 4; pSer473 and Figure 5; pThr308) and the downstream target TBC1D4 (Figure 6; pThr642) believed to act as a molecular switch for GLUT4 movement in the cell [20], [21], [27].

In contrast to our hypothesis, muscle-specific overexpression of kinase dead α2AMPK (AMPK KD) did not lead to insulin resistance with ageing. Furthermore, development of insulin resistance in response to high fat feeding was not exacerbated in old AMPK KD mice when compared to WT littermates. Using the same mouse strain, it was previously shown that high fat diet-induced insulin resistance is also not exacerbated in young AMPK KD mice [14], collectively supporting that functional AMPK is not protecting against development of high fat diet-induced insulin resistance in mouse muscle; at least not in AMPK KD mice on a C57BL/6J background.

Interestingly, both young and old AMPK KD mice on chow diet were slightly glucose intolerant, but had normal insulin tolerance, HOMA-IR values and insulin-stimulated glucose uptake in isolated muscle (Figure 1 and 2). In these mice the plasma insulin response during the OGTT was similar or lower (Figure 1B) despite higher blood glucose levels raising the possibility that lack of muscle AMPK activity may influence pancreatic function. Previously, normal glucose tolerance (1 g/kg body wt, 6 hours fasting) has been observed in young (6–9 weeks of age) AMPK KD mice [14] whereas adult (36 weeks of age) mice were slightly glucose intolerant (2 g/kg body weight, 12 hours fasting) [28]. Since glucose intolerance may be more easily masked at low glucose doses [29], the reason for these discrepancies likely relates to procedural differences. Furthermore, the apparent mildness of this metabolic phenotype in AMPK KD mice may further encumber experimental verification.

In the present study ageing per se did not result in development of insulin resistance as indicated by responses to OGTT, ITT and HOMA-IR (Figure 1). Also, both insulin signaling and insulin-stimulated glucose uptake were not impaired in muscle from old vs. young mice. Generally, aging is linked to insulin resistance in both humans and rodents [30], [31] and characterized by increased fat accumulation, chronic inflammation and oxidative stress in muscle [32], [33]. In the present study, although body weight increased with age, metabolic characteristics (RER, VO_2_) and importantly body composition were not markedly altered. Thus, our results indirectly support an important role of excessive fat accumulation as a key contributing factor in the etiology of age-induced insulin resistance, as previously speculated [30], [34].

When old mice were placed on a high fat diet for 17 weeks they became insulin resistant both at the whole body level and in skeletal muscle, coinciding with a ∼30% increase in body weight but unaltered lean body mass (Table 1). Despite excessive fat accumulation, these mice were normally glucose tolerant (Figure 1A), associated with a compensatory increase in circulating insulin concentration (Figure 1C). As described, lack of functional AMPK did not exacerbate the detrimental effect of high fat feeding. If anything AMPK KD mice exhibited slightly improved (although not significant) HOMA-IR values and ITT response compared to WT littermates (Figure 1C). Curiously, these observations are in stark contrast to a study of high fat feeding in mice overexpressing inactive α2AMPK bred on a FVB mouse background (AMPK Ki) [12]. In that study, high fat feeding led to a reduction in insulin-stimulated glucose uptake in WT mice, similar to our observations, whereas insulin-stimulated glucose uptake was abolished in AMPK Ki mice. Considering the implications of placing AMPK in the nexus of diet and insulin action, these contradicting observations deserve further consideration.

Despite different genetic approaches [15], [35] both mouse strains overexpress a non-functional α2AMPK isoform that displaces endogenous α2AMPK and to some extent α1AMPK. As a result, in both mouse strains, basal α2AMPK activity is markedly reduced, and activation of α2AMPK in response to pharmacological activators, hypoxia or muscle contraction is almost completely abolished [13], [15], [35], [36]. Based on the similarities in AMPK dysfunction, in our view, a plausible explanation for the different adaptations to high fat dieting may relate to the differences in mouse strains wherein kinase dead AMPK is induced. As an indication, the FVB strain of mice have previously been characterized as being more resistant to development of high fat diet-induced insulin resistance than mice on a C57BL/6J background [37]. Furthermore, it is noteworthy, that in muscle from high fat fed AMPK Ki mice on a FVB background, reduced protein content of a range of proximal insulin signaling components are observed including the insulin receptor β subunit (∼30%), IRS-1 (∼50%) and Akt (∼40%) when compared to WT or AMPK Ki mice on a chow diet [12]. Based on similar Akt2 protein content (Figure 8E) and insulin signaling (Figure 4, 5, 6) in the present study, similar adaptations in AMPK KD mice on a C57BL/6J background apparently do not take place.

In contrast, it should be emphasized that in our model both SOL and EDL muscle are characterized by significant reductions of TRB-3 protein content independent of age and diet (Figure 8F). TRB-3, the mammalian homolog of *drosophila tribbles*, is emerging as an important player in insulin signaling, by its capacity to bind to Akt and prevent phosphorylation in the activation loop (pSer473 and pThr308) [38]. In LKB-1 KO mice, reduced TRB-3 protein content has been suggested as a critical adaptation improving insulin-stimulated glucose uptake [39] and also more recently, improved insulin signaling and insulin-stimulated glucose uptake after exercise in *ob/ob* mice have been associated with exercise-induced reductions in TRB-3 protein content [40]. The present study indicates a role of AMPK to regulate expression of TRB-3 although by yet undefined mechanisms in mouse muscle. Furthermore, reduced TRB-3 expression in our AMPK KD model may contribute to explaining the normal insulin signaling and insulin-stimulated glucose uptake in our AMPK KD model.

Collectively, this study provides evidence that high fat diet-induced insulin resistance in mouse skeletal muscle on a C57BL/6J background is associated with impaired insulin signaling at the level of Akt and importantly also TBC1D4, providing a novel link between insulin signaling defects and impairments in control of GLUT4 translocation. In contrast to our hypothesis, the lack of functional AMPK did not influence insulin-stimulated glucose uptake with ageing or exacerbate insulin resistance after high fat feeding in old mice. Thus, based on elaborate studies of AMPK KD mice, the lack of α2AMPK activity in muscle (both oxidative and glycolytic) does not result in insulin resistance in neither lean nor obese, young or old mice ([14], present study). This strongly suggests that in contrast to previously indicated, AMPK does not constitute a necessary protective component for normal insulin action in muscle.
